# The (in)dependency of blood and sweat sodium, chloride, potassium, ammonia, lactate and glucose concentrations during submaximal exercise

**DOI:** 10.1007/s00421-020-04562-8

**Published:** 2020-12-23

**Authors:** L. Klous, C. J. de Ruiter, S. Scherrer, N. Gerrett, H. A. M. Daanen

**Affiliations:** 1 Department of Human Movement Sciences, Faculty of Behavioural and Movement Sciences, Amsterdam Movement Sciences, Vrije Universiteit Amsterdam, Amsterdam, The Netherlands; 2 Department of Health Sciences and Technology, Institute of Human Movement Sciences and Sport, ETH Zurich, Zurich, Switzerland

**Keywords:** Ammonia, Blood, Electrolytes, Glucose, Lactate, Sweat

## Abstract

**Purpose:**

To reduce the need for invasive and expensive measures of human biomarkers, sweat is becoming increasingly popular in use as an alternative to blood. Therefore, the (in)dependency of blood and sweat composition has to be explored.

**Methods:**

In an environmental chamber (33 °C, 65% relative humidity; RH), 12 participants completed three subsequent 20-min cycling stages to elicit three different local sweat rates (LSR) while aiming to limit changes in blood composition: at 60% of their maximum heart rate (HR_max_), 70% HR_max_ and 80% HR_max_, with 5 min of seated-rest in between. Sweat was collected from the arm and back during each stage and post-exercise. Blood was drawn from a superficial antecubital vein in the middle of each stage. Concentrations of sodium, chloride, potassium, ammonia, lactate and glucose were determined in blood plasma and sweat.

**Results:**

With increasing exercise intensity, LSR, sweat sodium, chloride and glucose concentrations increased (*P* ≤ 0.026), while simultaneously limited changes in blood composition were elicited for these components (*P* ≥ 0.093). Sweat potassium, lactate and ammonia concentrations decreased (*P* ≤ 0.006), while blood potassium decreased (*P* = 0.003), and blood ammonia and lactate concentrations increased with higher exercise intensities (*P* = 0.005; *P* = 0.007, respectively). The vast majority of correlations between blood and sweat parameters were non-significant (*P* > 0.05), with few exceptions.

**Conclusion:**

The data suggest that sweat composition is at least partly independent of blood composition. This has important consequences when targeting sweat as non-invasive alternative for blood measurements.

**Electronic supplementary material:**

The online version of this article (10.1007/s00421-020-04562-8) contains supplementary material, which is available to authorized users.

## Introduction

To reduce the need for invasive and expensive measures of human biomarkers, there is a considerable interest in the development of continuous and non-invasive measurement techniques that are commonly manifested in wearable sensors (Alizadeh et al. 2018; Gao et al. 2016, 2018; Heikenfeld et al. 2018). Currently, sweat is the most preferred bodily fluid to use for these non-invasive measurements, alternative to blood, urine and saliva. That is, because with appropriate sampling techniques it is possible to collect sweat easily from a large surface area. Despite the development of several wearable sweat sensors (Alizadeh et al. 2018; Gao et al. 2016), these advance far quicker than scientific knowledge on the physiological mechanisms determining sweat composition. Since extracellular fluid is the precursor for primary sweat in the coil of eccrine sweat glands (Kuno 1956), direct relations between blood and sweat composition are sometimes assumed. However, before eccrine sweat is released onto the skin surface, modifications to the primary solution are made during its passage along the sweat glands duct (Baker and Wolfe 2020). Due to such modifications, blood and sweat composition do not necessarily relate.

Considerable literature on relations between biomarkers in blood and sweat exists but results are inconclusive, potentially due to the limited number of participants (Bandodkar et al. 2019; Lee et al. 2016, 2017), different stimulation methods of sweat production (pilocarpine iontophoresis, passive heating and active heating), a variety of sweat collection methods (absorbent patches, arm bag technique, whole-body washdown), different skin preparation strategies (none, soap, alcohol, deionised water), variation in hydration level (combination of exercise duration and drinking ability), and/or absence of reporting statistical correlation analyses (Ament et al. 1997; Bandodkar et al. 2019; Czarnowski et al. 1992; Green et al. 2000; Hew-Butler et al. 2010; Lee et al. 2016, 2017). The experiments described in the available literature were also limited to measuring a maximum of three different components in sweat, which makes it difficult to get a bigger picture of the (in)dependency of blood and sweat composition. In the present study, relations between blood and sweat for a total of six commonly measured biomarkers (sodium, chloride, potassium, ammonia, lactate and glucose) were investigated. These six biomarkers are thought to reflect the biochemical environment of the human body and have the potential to be easily detected in sweat.

Previous research commonly elicited relatively large changes in blood composition (Alvear-Ordenes et al. 2005; Ament et al. 1997; Czarnowski et al. 1992; Green et al. 2000; McCubbin et al. 2019). With large changes in blood composition, it may become more likely to observe concomitant changes in sweat composition because precursor sweat originates from blood plasma. However, due to subsequent modifications in the sweat gland, such as the ductal reabsorption of sodium and chloride prior to sweat excretion onto the skin surface, blood and sweat composition may also become (partly) independent. That is why, in the present study, we are interested in potential relations between blood and sweat composition when changes in blood composition can expected to be limited. Considering the development of wearable sensors that continuously predict blood composition based on sweat composition, conditions with limited changes in blood composition will also occur more often in daily life and during submaximal exercise than those with large changes in blood composition, like during high-intensity anaerobic exercise or with intake of large amounts of sodium, chloride or ammonia (Alvear-Ordenes et al. 2005; Ament et al. 1997; Czarnowski et al. 1992; Green et al. 2000; McCubbin et al. 2019).

Sweat sodium and chloride are suggested to be potential biomarkers for an electrolyte imbalance, as in hyponatremia, muscle cramps and dehydration (Baker 2017; Morgan et al. 2004; Stofan et al. 2005). Extracellular fluid is a precursor to primary sweat, yet reabsorption of sodium and chloride in the sweat glands ducts is presumably independent of blood concentrations (Kirby and Convertino 1986). Sweat sodium concentrations most likely depend on local sweat rate (LSR), with higher LSR leading to increases in sweat sodium concentrations (Buono et al. 2008). This appears to be related to a disproportional increase in reabsorption rates compared to secretion rates of sodium in sweat. Since the channels that are responsible for sodium and chloride reabsorption have a functional interaction (Reddy and Quinton 2003), similar patterns may be observed for chloride in sweat. Sweat potassium, as the major cation in intracellular fluid, is also suggested to be a biomarker for an electrolyte imbalance. Primary sweat in the secretory coil is nearly isotonic to blood plasma and presumably potassium is not reabsorbed in the proximal duct (Kuno 1956; Robinson and Robinson 1954; Sonner et al. 2015). Ammonia is typically found in relatively high concentrations in human sweat and is thought to reflect blood concentrations (Alvear-Ordenes et al. 2005; Czarnowski et al. 1992). However, these earlier findings were observed in the presence of large disturbances in blood homeostasis due to anaerobic exercise or ingestion of large amounts of ammonia. Lactate in sweat is most likely a by-product of the eccrine sweat gland metabolism (Derbyshire et al. 2012), rather than originating from blood plasma. Metabolically, an increased sweat lactate concentration could be expected when LSR is higher. However, previous research showed that sweat lactate bares a proportionally inverse relation with LSR due to dilution (Ament et al. 1997; Buono et al. 2010; Derbyshire et al. 2012). Little is known about the functional role of glucose in the eccrine sweat gland, but there is a recent surge of interest for this metabolite in sweat (Bandodkar et al. 2018; Lee et al. 2017). Excretion of glucose in sweat is suggested to depend mainly on changes in blood glucose concentrations (Lee et al. 2016, 2017; Moyer et al. 2012).

The purpose of this study was to investigate whether relations between blood and sweat sodium, chloride, potassium, ammonia, lactate and glucose would exist within the ranges that are usually found during submaximal exercise in healthy individuals. The purpose of limiting changes in blood concentrations was to ensure that it remained within typical physiological range during submaximal exercise in healthy individuals. Since inducing sweat production is essential for wearable sweat monitoring and LSR affects sweat composition, LSR was manipulated by exercising at three incremental exercise intensities. Because it is likely that sodium and chloride reabsorption in the eccrine sweat glands are independent of blood concentrations, the sodium and chloride concentrations were expected to be unrelated between blood and sweat. Due to the absence of a reabsorption mechanism for sweat potassium, blood and sweat potassium were hypothesised to be significantly correlated. It is not known what happens to sweat ammonia concentrations when changes in blood ammonia are limited. Assuming that sweat ammonia concentrations also depend on blood ammonia under conditions of limited changes in blood, relations between blood and sweat ammonia were expected. Since sweat lactate concentrations seem to depend on LSR, rather than changes in blood lactate concentrations, it was hypothesised that blood and sweat lactate are not related. Lastly, relations between blood and sweat glucose were expected.

## Methods

### Ethical approval

Procedures were approved by the Medical Ethics Committee of Erasmus MC Rotterdam (MEC-2019-0202). The study was conducted in accordance with the guidelines of the revised *Declaration of Helsinki* (2013). Participants were informed of the study procedures and potential risks were identified, before written informed consent was obtained.

### Participants

Twelve individuals (nine males, three females; age = 25 ± 4 years; height = 181.0 ± 7.8 cm; body mass = 74.3 ± 10.7 kg; *V*O_2max_ = 52.2 ± 9.4 mL kg^−1^ min^−1^) participated in this study. Participants were all classified as ‘trained’ according to previously established criteria (De Pauw et al. 2013; Decroix et al. 2016). Participants were instructed to refrain from alcohol and strenuous exercise 24 h, and to avoid caffeine consumption 12 h prior to the experiment. To promote hydration, participants were asked to consume 500 mL of water the night before and another 500 mL prior to the experiment. No restrictions were placed on participants diets to not interfere with homeostasis and to represent a typical situation in which participants could wear a sweat-monitoring device with their normal sweating response. Participants filled out a food diary to check for abnormal intake of certain nutrients (i.e., the measured sweat components). This was not the case. All participants were non-smokers, did not take any prescription medication, had no history of heat-related illnesses, cardiovascular complications and did not have any known issues with thermoregulation. All female participants were taking oral contraceptives and were in the follicular phase of their menstrual cycle during the study.

### Study design

Participants reported to the laboratory twice. During the first visit maximum oxygen uptake (*V*O_2max_) and maximum heart rate (HR_max_) were determined during an incremental cycling test in temperate conditions (20 °C, 50% relative humidity; RH). They started cycling (Lode Excalibur, Groningen, The Netherlands) at 100 W for 3 min and after that power output increased by 25 W min^−1^ until volitional exhaustion. The second visit consisted of incremental cycling in a climate chamber (B-Cat, Tiel, The Netherlands) set to 33 °C and 65% RH. Since inducing sweat production is essential for wearable sweat monitoring and LSR affects sweat composition (Buono et al. 2007, 2010), LSR was manipulated. To minimise the potential direct dependency of sweat on blood composition (considering its origination from blood plasma), we aimed for limited changes in blood composition. To this end, participants cycled for 30 min at 60% HR_max_, 20 min at 70% HR_max_, 20 min at 80% HR_max_ and had 20 min of post-exercise seated-rest. The 30 min at 60% HR_max_ included a 10-min warming-up after 10 min of seated-rest, together allowing for a 20-min wash out period of the skin (Baker and Wolfe 2020) to prevent skin contamination. The specific exercise intensities were chosen based on pilot testing: 60% HR_max_ was the lowest intensity that elicited enough sweat for chemical analysis, whilst 80% HR_max_ was the highest intensity participants could successfully complete without showing considerable changes in blood values. Participants were not allowed to drink throughout the protocol.

### Instrumentation

To determine *V*O_2max_ and HR_max_, oxygen uptake was measured breath-by-breath using a gas analyser (Quark CPET, COSMED, Rome, Italy) and participants wore a HR monitor (Polar Vantage M, Kemele, Finland). For the second visit, participants ingested a telemetry pill (BodyCap, Hérouville-Saint-Clair, France) 6–8 h in advance for measuring gastrointestinal temperature (*T*_gi_) (Byrne and Lim 2007). Hydration status, as indicated by urine specific gravity ≤ 1.020 (Kenefick and Cheuvront 2012), was confirmed in all participants using a handheld refractometer (Atago Co. Ltd, Tokyo, Japan). Participants were equipped with four skin temperature sensors (i-Button 1922L, Maxim Integrated Products, San Jose, CA, USA) on the chest, forearm, thigh and calf, and a weighed mean skin temperature ($\stackrel{-}{T}$_sk_) was calculated (Ramanathan 1964). Participants wore a HR monitor throughout.

### Blood sampling

Prior to cycling in the heat, a cannula (Venflon, BD, New Jersey, USA) was inserted to a superficial antecubital vein of the arm while participants sat in a semi-supine position. From this cannula, three blood samples (3 mL heparin Vacutainer; 3 mL EDTA vacutainer; 3 mL oxalate fluoride vacutainer, BD, New Jersey, USA) were collected in the middle of each stage (60%, 70% and 80% HR_max_, post-exercise). These three specific types of vacutainers were required to determine all six components. Between collection times, the catheter was kept patent by flushing with a 0.9% saline solution (PosiFlush, BD, New Jersey, USA). Blood samples were immediately centrifuged (1800* g*, 5 min) to separate plasma from the cells. Plasma was pipetted and frozen at −20 °C until analysis. Concentrations of sodium, chloride, potassium, ammonia, lactate and glucose were determined by ion-selective electrodes (Cobas 6000, Roche Diagnostics, Almere, The Netherlands) or enzymatically (NH3L; LACT2; GLUC3, Roche Diagnostics, Almere, The Netherlands, Table 1).

**Table 1:** Characteristics of the analysis

Component	LOD (mmol L^−1^)	CV (%)	Sample volume (μL)
Sodium	0.4	< 1	15
Chloride	0.4	< 2.2	15
Potassium	0.2	1.0–1.3	15
Ammonia	0.0001	3.1	20
Lactate	0.2	< 3.6	2
Glucose_sweat_	–	–	2
Glucose_blood_	0.11	0.7	2

Glucose concentration in sweat was determined with a dynamic multiple reaction monitoring method (described in Supplemental Procedure), which is not a routine analysis and, therefore, characteristics are not known

*LOD* limit of detection, *CV* coefficient of variation

### Sweat sampling

LSR was determined gravimetrically using the absorbent patch technique (Morris et al. 2013). To prevent skin contamination, skin of the right dorsal upper arm and upper back, covering zones 13 and 19 (Gerrett et al. 2014), was cleansed with alcohol, deionised water and dried with gauze pads before application of the patches (Cutisoft, BSN Medical, Almere, The Netherlands, size: 25 cm^2^, absorbent capacity: ~ 2.0 g). The absorbent patch was covered with an impermeable fabric (Parafilm M, Bemis, Saint Louis, USA) and fixed to the skin using a porous adhesive on top (Fixomull stretch, BSN Medical, Almere, The Netherlands). Sweat samples were collected for ~ 15 min during each of the three exercise bouts and post-exercise, totaling four samples per site. To prevent skin contamination, the skin was cleaned with alcohol, deionised water and dried with gauze pads in between applications. The sampling period of ~ 15 min was chosen to collect enough sweat for chemical analysis but to prevent saturation. To prevent skin contamination and hidromeiosis underneath the patches, they were visually inspected and removed earlier if necessary. Patches were carefully replaced using marks on the skin. While applying each patch, the researcher wore gloves and only touched the absorbent with tweezers. Prior to and directly after application, each patch was weighed (1419MP8-1, Sartorius, Goettingen, Germany, accuracy: 1 mg) in a sealed plastic tube (Salivette, Sarstedt, Nümbrecht, Germany) to determine LSR according to

$$\mathrm{LSR}=\frac{\frac{{m}_{\mathrm{wet}}-{m}_{\mathrm{dry}}}{\mathrm{SA}}}{\mathrm{t}},$$

where ${m}_{\mathrm{wet}}$ refers to mass of the patch (mg) after the experiment, ${m}_{\mathrm{dry}}$ the dry mass of the patch (mg), SA surface area (cm^2^) and *t* application time (min). LSR is given in mg cm^−2^ min^−1^. The sealed tubes were centrifuged (1800* g* for 5 min) and frozen at −20 °C until analysis. Similar to blood plasma, concentrations of sodium, chloride, potassium, ammonia and lactate were determined (Table 1). Due to the very low concentrations of glucose in sweat, this metabolite was determined with a dynamic multiple reaction monitoring method (described in Supplemental Procedure). This is not a routine analysis and, therefore, accuracy of this method is not known. Due to logistical constraints, sweat samples from the upper arm could not be analysed for glucose.

### Statistical analysis

Statistical analyses were performed using IBM SPSS Statistics 26.0. Effects were considered significant if *P* < 0.05. Data are presented as means and standard deviations (SD). We used the Shapiro–Wilk test to check if the data were normally distributed. To assess whether concentrations differed between blood and sweat (arm and back) and to assess whether changes in HR, *T*_gi_, *T*_sk_, LSR and sweat composition occurred throughout the protocol, a repeated-measures ANOVA was used with medium (blood, sweat on the arm, sweat on the back) and exercise intensity (60%, 70% and 80% HR_max_, post-exercise) as within-subject factor. Violations of sphericity were corrected using the Greenhouse–Geisser adjustment. Bonferroni post hoc analyses were conducted relative to 60% HR_max_ and each previous stage. To assess the relation between blood and sweat composition, simple linear regressions and Pearson rank correlation coefficients were conducted between blood and sweat sodium, chloride, potassium, ammonia and lactate from the arm and back and glucose from the back. Prediction strength of these correlations was expressed via correlation coefficients (*r*). The strength of the relations was classified as very weak (*r* ≤ 0.3), weak (*r* = 0.3–0.5), moderate (*r* = 0.5–0.7) or strong (*r* > 0.7).

## Results

All participants completed the protocol. *V*O_2max_ and HR_max_ were, respectively, 52.2 ± 9.4 mL kg^−1^ min^−1^ and 191 ± 10 bpm. Due to a relatively low LSR for one of the female participants, sweat sodium, chloride and potassium concentrations could not be determined during 60% HR_max_ on the arm and during post-exercise on the arm and back. The incremental cycling in the heat induced gradual increases in physiological responses (Table 2).

**Table 2:** Mean (SD) of the physiological parameters during incremental cycling in the heat (33 °C, 65% RH; *n* = 12)

Parameter	60% HR_max_	70% HR_max_	80% HR_max_	Post-exercise
HR (bpm)	122 (3)	135 (9)^a,^^b^	148 (9)^a,^^b^	121 (11)^b^
%HR_max_	64 (4)	71 (4)^a,^^b^	77 (8)^a,^^b^	–
*T*_gi_ (°C)	37.2 (0.1)	37.6 (0.1)^a,^^b^	38.0 (0.1)^a,^^b^	38.2 (0.1)^a,^^b^
$\stackrel{-}{{\varvec{T}}}$_sk_ (°C)	36.0 (0.3)	36.2 (0.1)^a,^^b^	36.4 (0.1)^a,^^b^	36.6 (0.1)^a,^^b^
LSR_arm_ (mg cm^−2^ min^−1^)	1.5 (0.6)	2.0 (0.8)^a,^^b^	1.9 (0.6)	1.7 (0.8)
LSR_back_ (mg cm^−2^ min^−1^)	2.1 (0.7)	2.4 (0.7)^a,^^b^	2.5 (0.8)^a^	1.8 (0.7)^a,^^b^

*HR* heart rate, *%HR*_*max*_ % of maximum heart rate, *T*_*gi*_ gastrointestinal temperature, $\stackrel{-}{T}$_*sk*_ weighed mean skin temperature, *LSR* local sweat rate

^a^*P* < 0.05 from 60% HR_max_

^b^*P* < 0.05 from the previous stage

### Comparison of blood and sweat composition

**Fig. 1:**
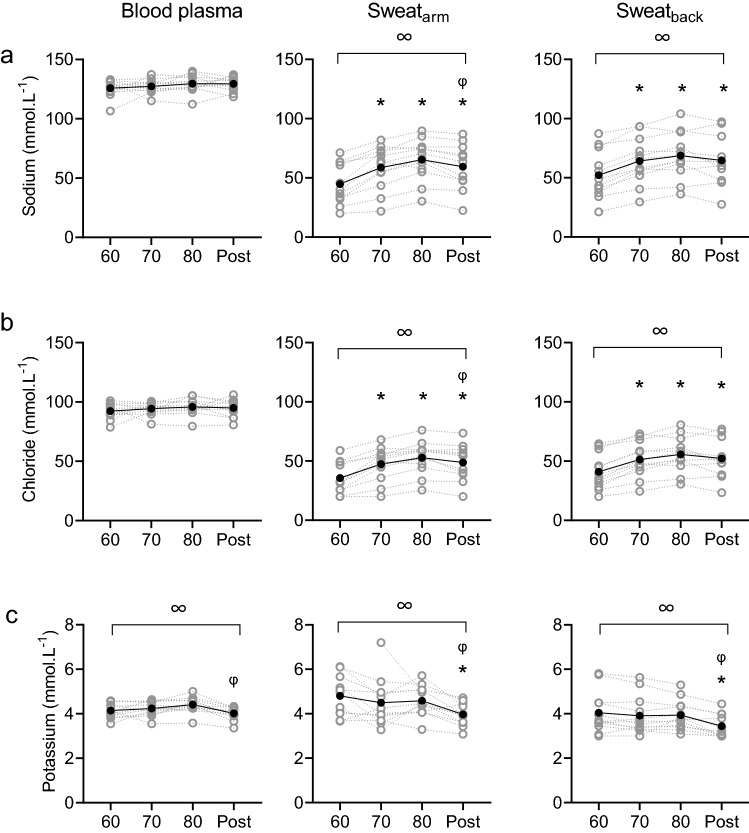

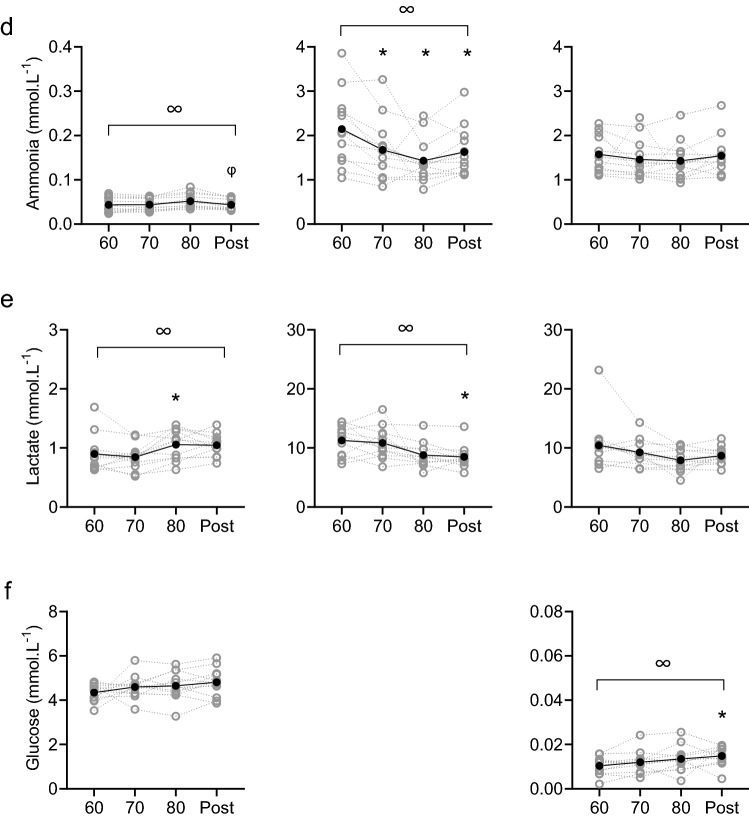
Sodium (**a**), chloride (**b**), potassium (**c**), ammonia (**d**), lactate (**e**) and glucose (**f**) concentrations in blood plasma (left panels), upper arm sweat (central panels) and upper back sweat (right panels) during incremental cycling (60%, 70%, 80% HR_max_) and post-exercise (post) in the heat (33 °C, 65% RH; *n* = 12). Grey symbols and lines represent individual data. Black symbols and lines represent group averages. Sweat samples on the arm were not analysed for glucose. Note that axes for blood and sweat ammonia, lactate and glucose differ. ∞ denotes significant (*P* < 0.05) main effects of exercise intensity. * denotes significant differences from 60% HR_max_. φ denotes significant differences from 80% HR_max_

Overall concentrations of sweat sodium and chloride on the arm and back, and glucose on the back were lower than in blood (*P* < 0.001), whilst concentrations of ammonia and lactate were consistently higher in sweat compared to blood (*P* < 0.001; Fig. 1). Sweat potassium concentrations on the upper back were also significantly lower than in blood (*P* = 0.011), but potassium concentrations in upper arm sweat were similar to blood plasma (*P* = 0.134; Fig. 1). Furthermore, sweat sodium, chloride, potassium and ammonia concentrations were higher on the arm compared to the back (*P* < 0.001; Fig. 1). Sweat lactate concentrations were similar on both measurement locations (*P* = 0.990; Fig. 1).

### Blood and sweat composition with increasing exercise intensity

Although we aimed to limit changes in blood, there were significant main effects of exercise intensity on blood potassium (*P* = 0.003), ammonia (*P* = 0.005) and lactate concentrations (*P* = 0.007), but not for sodium (*P* = 0.382), chloride (*P* = 0.365) and glucose concentrations in blood (*P* = 0.093; Fig. 1). Post hoc analyses revealed that relative to 80% HR_max_, significant post-exercise decreases were observed for blood potassium (*P* = 0.003) and ammonia (*P* = 0.019), whilst blood lactate concentrations were higher in 80% HR_max_ than 60% HR_max_ (*P* = 0.019) (Fig. 1).

There were significant main effects of exercise intensity on sweat sodium (arm: *P* < 0.001; back: *P* < 0.001), chloride (arm: *P* < 0.001; back: *P* < 0.001), potassium (arm: *P* < 0.001; back: *P* = 0.001), ammonia (arm: *P* = 0.006, back: *P* = 0.628), lactate (arm: *P* = 0.001, back: *P* = 0.066) and glucose concentrations (back: *P* = 0.026; Fig. 1). Post hoc analyses revealed that relative to 60% HR_max_ sweat sodium, chloride and ammonia (only on the arm) concentrations were higher during each stage (*P* ≤ 0.030). In addition, sweat potassium and lactate were significantly reduced (*P* ≤ 0.010; *P* ≤ 0.023, respectively) and glucose was significantly elevated post-exercise (*P* = 0.046; Fig. 1).

### Correlations between blood and sweat composition

**Fig. 2:**
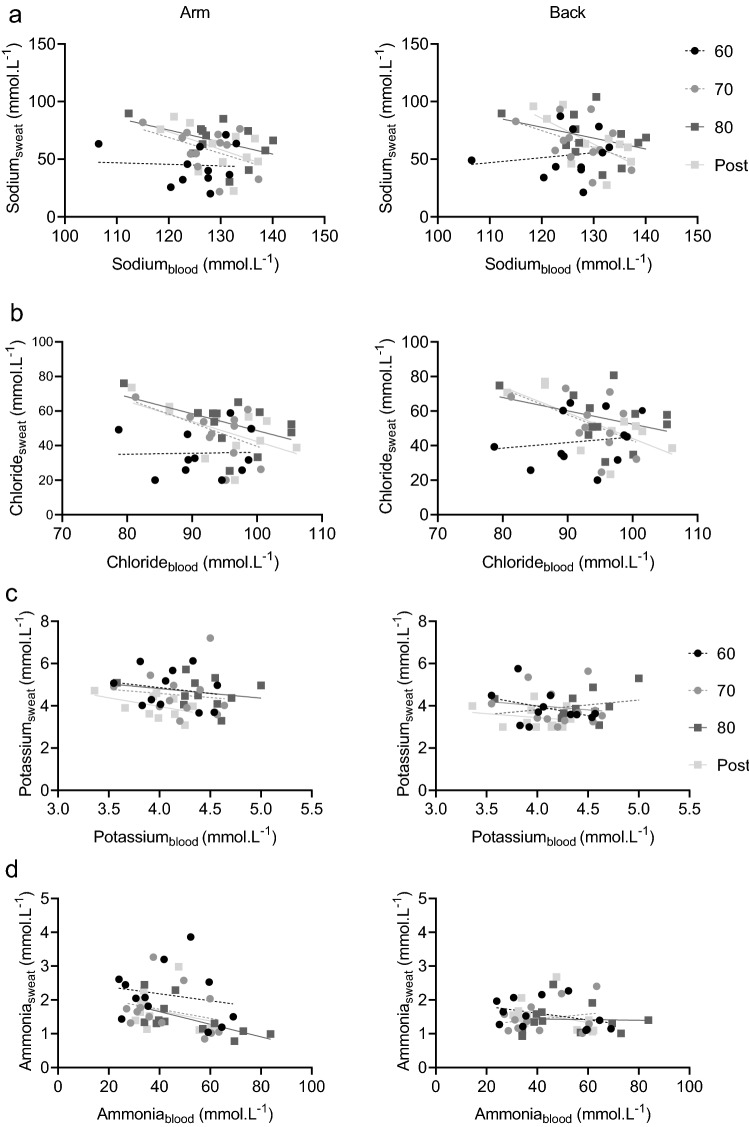

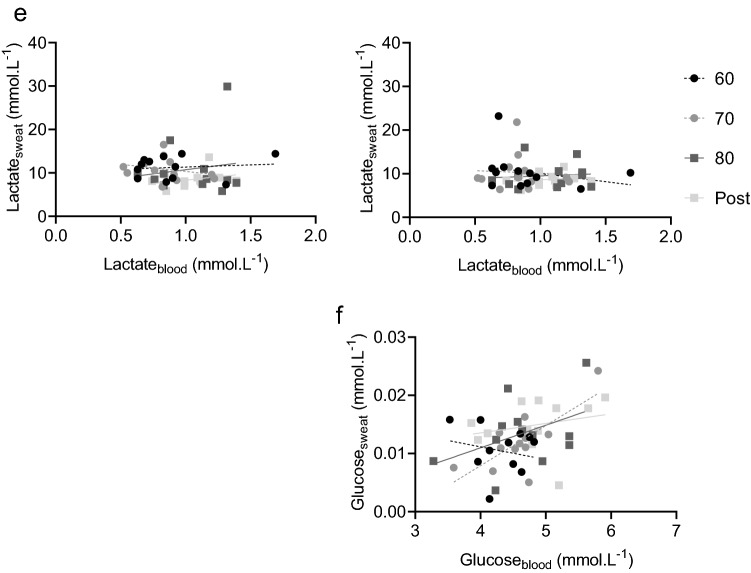
Correlations for sodium (**a**), chloride (**b**), potassium (**c**), ammonia (**d**), lactate (**e**) and glucose (**f**) concentrations in blood plasma and upper arm sweat (left panels) and upper back sweat (right panels) during incremental cycling in the heat (33 °C, 65% RH; *n* = 12). Black circles with and a dashed regression line represent 60% HR_max_ (60), grey circles and a dashed line represent 70% HR_max_ (70), dark grey squares and a solid line represent 80% HR_max_ (80) and light grey squared and a solid line represent post-exercise (post). Corresponding correlation coefficients can be found in Supplemental Table 1

The majority of correlations between blood and sweat composition were non-significant: 19 out of 20 on the arm (5 components × 4 exercise intensities) and 21 out of 24 on the back (6 components × 4 exercise intensities; Fig. 2). All the correlation coefficients for each component at each exercise intensity on the arm and back can be found in Supplemental Table 1. To summarise, blood sodium was non-significantly correlated with sweat sodium from the arm and back at all exercise intensities (*r* ≤ −0.50, *P* > 0.05). The exception to this was at the back, where there was a moderate negative relation between blood and sweat sodium post-exercise (*r* = −0.67, *P* = 0.025; Fig. 2). A similar finding for blood and sweat chloride was observed, with no significant correlations at both locations and any of the exercise intensities (*r* ≤ −0.57, *P* > 0.05). The exception to this was at the back post-exercise, where a moderate negative relation was observed (*r* = −0.68, *P* = 0.020; Fig. 2). Correlations between blood and sweat potassium on the arm and back were non-significant throughout the protocol (*r* ≤ −0.46, *P* > 0.05; Fig. 2). Blood and sweat ammonia were not related at any of the exercise intensities for both locations (*r* ≤ −0.38, *P* > 0.05), with the exception of the upper arm during exercise at 80% HR_max_ (*r* = −0.62, *P* = 0.003; Fig. 2). There were no significant correlations between blood and sweat lactate at any exercise intensity for both locations (*r* ≤ 0.31, *P* > 0.05; Fig. 2). Lastly, there was a strong positive relation between blood and upper back sweat glucose during exercise at 70% HR_max_ (*r* = 0.73, *P* = 0.007), but at all other exercise intensities, significant correlations were absent (*r* ≤ 0.43, *P* > 0.05; Fig. 2).

## Discussion

Although previous research related single biomarkers in blood to sweat, results are inconclusive, potentially due to methodological limitations (as mentioned in “Introduction”). The added value of the present study is that we determined six commonly measured biomarkers in blood and sweat during exercise from 12 healthy individuals in a single experiment using the common absorbent patch technique for sweat collection. Moreover, relations between blood and sweat were examined under submaximal exercise conditions where limited changes in blood composition can be expected. This was done because with precursor sweat originating from blood plasma, large changes in blood composition are potentially reflected in sweat, whilst most of the time (i.e., in daily life and during submaximal exercise) changes in blood composition are limited. Sweat composition was successfully manipulated by gradually increasing LSR, whilst changes in blood were limited for three of the six components that were analysed (sodium, chloride and glucose). The vast majority of correlations between blood and sweat composition were non-significant, with few exceptions. Taken together, such findings show that sweat composition is at least partly independent of blood composition. This has to be taken into consideration when aiming to use sweat as non-invasive alternative for blood measurements during exercise.

### LSR

As expected, LSR on the arm and back increased from 60% HR_max_ to 70% HR_max_ (Table 2). The increase in LSR is most likely due to increased metabolic heat production (Cramer and Jay 2016). This idea is supported by the simultaneous increase in *T*_gi_ and *T*_sk_. Interestingly, LSR on the arm and back did not rise further from 70% HR_max_ to 80% HR_max_. Considering the continuous increase in *T*_gi_ and *T*_sk_, which are the drivers for sweat production (Nadel et al. 1971a, b), an increase in LSR was expected. It could be that a steady-state sweat rate had been achieved during the protocol, explaining the absence of a further rise in LSR. Previous research observed a steady-state sweat rate after 30–40 min of cycling (Gagnon et al. 2013; Ravanelli et al. 2020). Since the 80% HR_max_ stage corresponds to min 50–70 of cycling, reaching a steady-state sweat rate seems a rational possibility. Second, sweat loss without the ability to drinks leads to dehydration (Strydom et al. 1966). Reduced plasma volume as a result of dehydration (3% and 5% body mass loss) is known to impair LSR (Fortney et al. 1981; Nadel 1985; Sawka et al. 1985). We had a slight reduction in blood plasma due to blood sampling (~ 50 mL in total) and body mass loss only ranged from 1.1% to 3.0%. Therefore, dehydration and the concomitant consequences on blood volume seem unlikely to explain LSR outcomes. Lastly, the decrease in LSR on both measurement locations from 80% HR_max_ to post-exercise rest can be explained by the decreased metabolic heat production.

### Sodium and chloride

Alongside the increases in LSR, sweat sodium and chloride increased as exercise intensity increased. The elevation in sweat sodium occurs when the rate of sodium secretion exceeds the reabsorption rate in the eccrine sweat gland. As LSR increases, the secretion becomes disproportionally higher, potentially due to insufficient time for reabsorption to occur (Buono et al. 2008). Sweat sodium and chloride concentrations were lower than blood plasma concentrations, which is in accordance with previous research (Kuno 1956; Robinson and Robinson 1954; Sonner et al. 2015). The presumable underlying mechanism of this discrepancy is the ductal reabsorption of sodium and chloride from nearly isotonic precursor sweat (Kuno 1956). The reabsorption rate is thought to depend on LSR rather than blood composition (Buono et al. 2008; Kirby and Convertino 1986), explaining why we observed increases in sweat sodium and chloride in the presence of limited changes in blood composition. Obviously, as concentrations in sweat increased whilst simultaneously blood concentrations did not change significantly, no relations between blood and sweat composition were found. Our data are in line with previous research reporting no significant correlations between blood and sweat for sodium and chloride. These studies either included a small sample size (*n* = 3) (Johnson et al. 1944), collected sweat by the non-commonly used arm bag technique (hand and forearm inserted in plastic bag to collect sweat) (Johnson et al. 1944; Mickelsen and Keys 1943) or collected blood and sweat pre- and post-exercise rather than during exercise (McCubbin et al. 2019). With McCubbin et al. (2019) already reporting the absence of relations pre- and post-exercise in a larger group of participants (*n* = 15) using the common absorbent patch technique for sweat collection, our data add to knowledge on this topic by showing that also during submaximal exercise the majority of sweat sodium and chloride concentrations do not significantly correlate with blood concentrations.

### Potassium

First, the observed potassium concentrations were within the range (2–8 mmol L^−1^) of previously reported research (Baker and Wolfe 2020). This suggests that we limited skin contamination, which is typically indicated by unusual high concentration (> 8 mmol L^−1^) of sweat potassium (Weschler 2008). As expected, concentrations of blood and upper arm sweat potassium were comparable (Kuno 1956; Robinson and Robinson 1954; Sonner et al. 2015). Contrarily, potassium concentrations in upper back sweat were lower compared to blood plasma, which may be explained by the observation that sweat potassium on the back is less concentrated than on extremities (Baker et al. 2019). Since skeletal muscles are the major reservoir for potassium storage in the human body, exercise causes considerable increases in blood potassium concentrations (Medbo and Sejersted 1990). The decrease in blood potassium concentration following exercise may be caused by removing the exercise stimuli, and was reflected by significantly lower sweat potassium concentrations. Such a finding suggests that changes in blood plasma concentrations contribute to changes in sweat potassium concentration, rather than LSR changes. This is presumably due to the lack of a reabsorption mechanism for potassium in the eccrine sweat glands ducts (Kuno 1956; Robinson and Robinson 1954; Sonner et al. 2015). However, for potassium no significant correlations were observed between blood and sweat, probably because the simultaneous drop in blood and sweat potassium only occurred for one datapoint (post-exercise). Our findings support the one study we are aware of that found no relation between post-exercise blood and lower back sweat potassium that was collected during exercise (Vairo et al. 2017). Our data add that when blood and sweat are collected simultaneously during exercise for upper arm (preferred location for wearables) and upper back sweat, relations with blood potassium were not present either. The underlying mechanism remains unknown.

### Ammonia

In this study, sweat ammonia concentration was ~ 400 times higher compared to concentrations in blood and sweat ammonia decreased as exercise intensity increased (Fig. 1), which both correspond to previous research (Alvear-Ordenes et al. 2005; Ament et al. 1997; Zoerner et al. 2017). The discrepancy between ammonia concentrations in blood and sweat suggests that each modality has its own source. Lowenstein (1990) provided evidence indicating that ammonia in blood originates from the anaerobic glycolysis in active muscle fibers in which adenosine 5-monophosphate is degraded to inosine 5-monophosphate and ammonia in the myoadenylate deaminase reaction. This predominantly occurs during high-intensity exercise that was avoided in the present study. Sweat ammonia, on the other hand, is suggested to have multiple sources: eccrine sweat production (Brusilow and Gordes 1968), a by-product of the urea catabolism (Mosher 1933), formation in the skin (Itoh and Nakayama 1952), skin gas emission (Nose et al. 2005) and origination from blood plasma (Czarnowski et al. 1992). We found no relation between blood and sweat ammonia concentrations. This is in contrast with previous research reporting relations between blood and sweat ammonia when blood ammonia concentrations were significantly elevated by oral ingestion (Czarnowski et al. 1992) or high-intensity exercise (Alvear-Ordenes et al. 2005). These relations may have been observed because blood ammonia partly contributes to sweat ammonia. However, in the present study we show that sweat ammonia can also be independent of blood ammonia concentrations. This was indicated by the lack of relations and the inverse direction of change in blood and sweat ammonia: there was a significant decrease of sweat ammonia concentrations on the arm during exercise, whilst LSR was elevated and ammonia in blood even tended (non-significant) to increase during exercise. Post-exercise, sweat ammonia concentration significantly increased on the arm in the presence of a significant decrease in blood ammonia. It should also be noted that Czarnowski et al. (1992) used pilocarpine stimulation rather than exercise and that Alvear-Ordenes et al. (2005) used a combination of exercise and sauna bathing and utilised a 60-min sweat collection period. The methodological discrepancies between those studies and the present study may attribute to the contradictory conclusions. Furthermore, ammonia concentrations in upper back sweat showed the same pattern over time as for the arm but changes were non-significant. It could be that because back sweat is already more diluted than arm sweat, changes were smaller compared to the arm. However, no scientific evidence backs up this theory.

### Lactate

Unlike the other components, concentrations of sweat lactate on the arm and back were similar to each other, potentially due to large variation in concentrations. As LSR increased, sweat lactate concentrations on the arm were found to decrease. This likely is explained by dilution (i.e., lower lactate concentrations once LSR gets higher) (Buono et al. 2010). In addition, overall sweat lactate concentrations considerably exceeded blood lactate concentrations, which can be explained by the origin of sweat lactate: a metabolic by-product of the sweat gland (Derbyshire et al. 2012). In contrast to sweat, blood lactate concentrations slightly increased during the protocol. This is probably due to an increased muscle metabolism, releasing lactate to the blood stream. Decreases in sweat lactate with simultaneous increases in blood lactate concentrations confirm that sweat lactate is partly, or may even be entirely, independent of blood lactate concentrations. Our findings support previous well-controlled studies with a similar outcome (Alvear-Ordenes et al. 2005; Ament et al. 1997; Green et al. 2000). The innovative aspect here is that lactate in blood and sweat were collected simultaneously and on anatomically close skin sites (antecubital vein and upper arm). Like with ammonia, lactate concentrations in upper back sweat showed a decreasing pattern as on the arm but changes were relatively small.

### Glucose

In this study, sweat glucose concentrations were ~ 100 times lower compared to blood glucose concentrations. Interestingly, sweat glucose concentrations increased with a higher LSR, but the changes in blood glucose were non-significant. Based on previous reported relations, blood glucose is thought to be the primary source for sweat glucose (Bandodkar et al. 2019; Lee et al. 2016, 2017; Moyer et al. 2012). However, these studies focused on the development of a wearable sensor and only included a small sample size (*n* = 2–5) to collect sweat for device-testing with less methodological details on the physiological part of the experiments and the corresponding statistical analysis (Bandodkar et al. 2019; Boysen et al. 1984; Lee et al. 2016, 2017; Nyein et al. 2019). To our knowledge, one well-controlled physiological experiment has been performed to compare blood and sweat glucose (Moyer et al. 2012), but their participants were diabetic patients. In line with our findings, Silvers et al. (1928) reported non-significant correlations between blood and sweat glucose during passive heating, rather than exercise-induced sweating, when blood was measured before and after sweat sampling. Considering the relatively large size and polarity of the glucose molecule, it indeed seems unlikely that glucose molecules diffuse from blood to sweat through the small lumen of the sweat glands in healthy individuals (Baker and Wolfe 2020), especially without any established specific glucose channels. Our data add to knowledge on this topic by reporting the absence of relations between blood and sweat glucose during exercise in a group of 12 healthy participants.

## Limitations

A methodological limitation to the present study could be that we did not establish steady-state sweat rates during each exercise stage (60%, 70% and 80% HR_max_). It would have been interesting to evaluate sweat composition during steady-state sweat rates, since sweat rate is known to affect sweat composition (Buono et al. 2008). Recently, a pre-determined threshold for LSR (< 0.01 mg cm^−2^ min^−1^) was used to determine steady state (Ravanelli et al. 2020). For such determinations, a very accurate and continuous measure of LSR using ventilated capsules is needed. This was not feasible in the present study. The time to reach steady state has been reported to exceed 30 min (Gagnon et al. 2013; Ravanelli et al. 2020). To be able to safely complete the protocol used in the present study (60–80% HR_max_ in 33 °C and 65% RH), three separate cycling sessions should have been utilised that each included 50-min cycling stages (at least 30 min to reach steady-state followed by 20 min for blood and sweat sampling). Post-hoc evaluation of the LSR outcomes revealed that during 80% HR_max_, potentially a steady-state sweat rate was reached as the LSR difference from 70% HR_max_ was only 0.1 mg cm^−2^ min^−1^ in 20 min, corresponding to a minutely change of 0.005 mg cm^−2^ min^−1^. During 80% HR_max_, there were no significant correlations except for ammonia on the arm (Supplemental Table 1). Such findings suggest that even during steady state, correlations between blood and sweat may not be significant. However, future research should investigate this properly.

The rationale for including males and females in the present study is based on the fact that LSR is the main determinate of electrolyte concentrations, not sex. Baker et al. (2018) reported higher whole-body sweat rates, LSR and higher sweat sodium and chloride concentrations in males. The authors suggest that the higher concentrations of sodium and chloride relate to the higher sweat rates, rather than a true sex effect. This hypothesis is supported by Buono et al. (2008), showing that the sodium secretion rate increases proportionally more than the sodium reabsorption rate with increases in LSR. Another study concluded that most variations in the rate at which sodium is lost via sweat secretion can be explained by a flow-dependent relationship rather than a sex effect (van den Heuvel et al. 2007). One study reported slightly lower potassium concentrations in males compared to females (Lobeck and Huebner 1962). However, this was observed in the presence of higher sweat rates in males. We are aware of one study suggesting there is no effect of sex on sweat lactate and ammonia concentrations (Meyer et al. 2007). These concentrations were not corrected for LSR, but at least for lactate an inverse relation between LSR and lactate concentrations has been established (Buono et al. 2010). We have not identified research comparing sweat glucose concentrations in males and females. In conclusion, there is limited evidence to suggest the absence of a true sex effect on sweat composition and, therefore, our results are not expected to be affected by sex.

Furthermore, sweat was sampled from two skin sites (upper arm and upper back) in a relatively homogenous group regarding age (25 ± 4 years). Since the regional distribution of sweating likely changes with age (Inoue and Shibasaki 1996; Inoue et al. 1999), it is not known whether similar findings would be observed in older individuals.

In the present study, blood was taken from a superficial antecubital vein whilst sweat was sampled from both the arm and back. Relations between blood from the arm and sweat from the arm and back were investigated. Since several studies suggest that specific components diffuse from blood to sweat (Czarnowski et al. 1992; Lee et al. 2016; Moyer et al. 2012; Robinson and Robinson 1954), it would have been interesting to investigate relations between upper back sweat composition and blood from a closer skin site as well. However, sampling blood from the upper back was not feasible.

For glucose determinations in sweat, we used a dynamic multiple reaction monitoring method. We used this method because glucose concentrations in sweat are too low to be detected by commonly used analysis for blood and urine (for example, GLUC3, Roche Diagnostics, Almere, The Netherlands). The dynamic multiple reaction is not a routine analysis and, therefore, we cannot quantify the limit of detection and coefficient of variation of this method. Since changes in sweat glucose were also very limited, future research should consider the validity and reliability of this method.

## Conclusions

Whilst elevating local sweat rate but limiting changes in blood composition, the vast majority of correlations between blood and sweat composition was found to be non-significant. The present study shows that the composition of submaximal exercise-induced sweat is at least partly independent of blood composition (for sodium, chloride, potassium, ammonia, lactate and glucose). This has to be taken into consideration when aiming for sweat as a non-invasive alternative for blood measurements in daily life and during exercise below the anaerobic threshold. It is important to note that our findings only apply to the exercise intensities (60%, 70%, 80% HR_max_ and post-exercise) and two measurement locations (upper arm and upper back) utilised in the present study.

## Electronic supplementary material

Below is the link to the electronic supplementary material.

Supplementary file1 (DOCX 24 KB)

Supplementary file2 (DOCX 29 KB)

## Abbreviations

ANOVA: Analysis of variance
CV (%): Coefficient of variation
HR (bpm): Heart rate
LSR (mg cm^−^^2^ min^−^^1^): Local sweat rate
LOD (mmol L^−^^1^): Limit of detection
*m* (mg): Mass
*r*: Correlation coefficient
RH (%): Relative humidity
SA (cm^2^): Surface area
*t* (min): Time
*T*_gi_ (°C): Gastrointestinal temperature
$\overline{T}_{{{\text{sk}}}}$ (°C): Mean skin temperature
*V*O_2max_ (mL kg^−^^1^ min^−^^1^): Maximum oxygen uptake
