# Supplementary material for: The (in)dependency of blood and sweat sodium, chloride, potassium, ammonia, lactate and glucose concentrations during submaximal exercise
Source: Eur J Appl Physiol. 2020 Dec 23;121(3):803–16. doi: 10.1007/s00421-020-04562-8 (PMC7892530; doi:10.1007/s00421-020-04562-8)
Supplement: Supplementary file 1 — Supplementary file1 (DOCX 24 KB) [file 421_2020_4562_MOESM1_ESM.docx]

## Electronic Supplementary Material

## Supplemental Procedure: Sweat glucose analysis

## Sweat samples were frozen in 2 mL Supelco amber low absorbance vials (Sigma 29653-U, Sigma-Aldrich, Saint Louis, USA) and evaporated overnight in an Alpha 1-4 Freeze dryer (Martin Christ Gefriertrocknungsanlagen GmbH, Osterode am Harz, Germany).

## Oximation was performed by resuspending the dried samples in 50 µL MOX (~Methoxyamine-HCl, Sigma 89803, Sigma-Aldrich, Saint Louis, USA) 20 mg.mL^-1^ pyridine. After 5 sec vortexing samples were incubated at 30 °C for 90 min.

Silylation was performed by the addition of 80µL MSTFA (~N-Methyl-N-(trimethylsilyl)trifluoroacetamide, Thermo Scientific 11786957, Fisher Scientific, Landsmeer, The Netherlands) +1% TMCS (~Trimethylchlorosaline, Interscience, TS-88530, Breda, The Netherlands). After 5 sec vortexing, samples were incubated at 70 °C for 50 min.

After cooling, the derivatised samples were centrifuged for 1 min at 6000 rpm. Inserts with bottom-spring were placed in the vials and the supernatant was transferred to the insert.

2 µL of the supernatant was analysed by GC-MS (Agilent 7890B GC coupled to Agilent 7000D Mass Selective Detector, Agilent Technologies, The Netherlands) operated under the following conditions: carrier gas, helium; flow rate, 1.0 mL.min^-1^; column Zebron 7HG-G004-11 ZB-50 30m x 250 µm x 0.25 µm, Phenomenex; inlet temperature, 250 °C; injection mode, solvent vent; emission 35 µA, ionisation voltage, 70 eV; ion source temperature, 230 °C; quadrupole source temperature, 150 °C. After a solvent delay of 1 min the oven temperature was increased at 10 °C.min^-1^ to 190 °C, followed by 2 °C.min^-1^ to 220 °C, 5 °C.min^-1^ to 240 °C, 10 °C.min^-1^ to 260 °C and 30 °C minute^-1^ to 320 °C. Sweat glucose was acquired with a dynamic multiple reaction monitoring method.
