# Supplementary material for: The (in)dependency of blood and sweat sodium, chloride, potassium, ammonia, lactate and glucose concentrations during submaximal exercise
Source: Eur J Appl Physiol. 2020 Dec 23;121(3):803–16. doi: 10.1007/s00421-020-04562-8 (PMC7892530; doi:10.1007/s00421-020-04562-8)
Supplement: Supplementary file 2 — Supplementary file2 (DOCX 29 KB) [file 421_2020_4562_MOESM2_ESM.docx]

## Electronic Supplementary Material

## Supplemental Table 1. Pearson correlations of blood (from a superficial antecubital vein) and sweat (upper arm and upper back) composition during incremental cycling in the heat (33 °C and 65% RH) (*n* = 12). Glucose concentrations were not analysed in sweat from the upper arm. Sweat sodium, chloride and potassium concentrations in 60% HR_max_ and post-exercise include *n* =11.

|  | Arm | | | | | | Back | | | | | |
| --- | --- | --- | --- | --- | --- | --- | --- | --- | --- | --- | --- | --- |
|  | **Sodium** | **Chloride** | **Potassium** | **Ammonia** | **Lactate** | **Glucose** | **Sodium** | **Chloride** | **Potassium** | **Ammonia** | **Lactate** | **Glucose** |
| 60% HR_max_ | -0.06 | 0.03 | -0.20 | -0.20 | 0.12 | - | 0.16 | 0.14 | -0.37 | -0.38 | -0.27 | -0.21 |
| 70% HR_max_ | -0.45 | -0.51 | -0.12 | -0.27 | -0.26 | - | -0.44 | -0.52 | -0.15 | 0.23 | -0.06 | 0.73^b^ |
| 80% HR_max_ | -0.45 | -0.50 | -0.24 | -0.62^a^ | 0.15 | - | -0.36 | -0.36 | 0.25 | -0.04 | 0.10 | 0.43 |
| Post- exercise | -0.50 | -0.57 | -0.46 | -0.35 | 0.31 | - | -0.67^a^ | -0.68^a^ | -0.21 | -0.37 | 0.19 | 0.24 |

%HR_max_: % of maximum heart rate.

Values represent correlation coefficients (r): ^a^ P < 0.05, ^b^ P < 0.01
